# The role of coronary artery disease in lung transplantation: a propensity-matched analysis

**DOI:** 10.1007/s00392-024-02445-y

**Published:** 2024-04-08

**Authors:** Enzo Lüsebrink, Nils Gade, Paula Seifert, Felix Ceelen, Tobias Veit, Fabian Fohrer, Sabine Hoffmann, Julia Höpler, Leonhard Binzenhöfer, Daniel Roden, Inas Saleh, Hugo Lanz, Sebastian Michel, Christian Schneider, Michael Irlbeck, Roland Tomasi, Rudolf Hatz, Jörg Hausleiter, Christian Hagl, Christina Magnussen, Benjamin Meder, Sebastian Zimmer, Peter Luedike, Andreas Schäfer, Martin Orban, Katrin Milger, Jürgen Behr, Steffen Massberg, Nikolaus Kneidinger

**Affiliations:** 1 Department of Medicine I, LMU University Hospital, LMU Munich, Munich, Germany; 2 DZHK (German Center for Cardiovascular Research), Partner Site Munich Heart Alliance, Munich, Germany; 3 Department of Medicine V, Comprehensive Pneumology Center (CPC-M), German Center for Lung Research (DZL), LMU University Hospital, LMU Munich, Munich, Germany; 4 Institute for Medical Information Processing, Biometry, and Epidemiology, Ludwig-Maximilians-Universität München, Munich, Germany; 5 Department of Cardiac Surgery, LMU University Hospital, LMU Munich, Munich, Germany; 6 Division for Thoracic Surgery, LMU University Hospital, LMU Munich, Munich, Germany; 7 Department of Cardiology, University Heart and Vascular Center Hamburg, Hamburg, Germany; 8 DZHK (German Center for Cardiovascular Research), Partner Site Hamburg/Kiel/Luebeck, Hamburg, Germany; 9 Department of Cardiology, Angiology, and Pneumology, University Hospital Heidelberg, Heidelberg, Germany; 10 Department of Internal Medicine II, Heart Center Bonn, University Hospital Bonn, Bonn, Germany; 11 Department of Cardiology and Vascular Medicine, University Hospital Essen, University Duisburg-Essen, West German Heart- and Vascular Center, Essen, Germany; 12 Department of Cardiology and Angiology, Hannover Medical School, Hannover, Germany; 13 Department of Anesthesiology, LMU University Hospital, LMU Munich, Munich, Germany; 14 DZHK (German Center for Cardiovascular Research), partner site Heidelberg, Heidelberg, Germany

**Keywords:** Lung transplantation, Coronary artery disease, Transplant candidate selection, Cardiovascular evaluation, Extracorporeal membrane oxygenation, Revascularization

## Abstract

**Background and aims:**

Candidate** s**election for lung transplantation (LuTx) is pivotal to ensure individual patient benefit as well as optimal donor organ allocation. The impact of coronary artery disease (CAD) on post-transplant outcomes remains controversial. We provide comprehensive data on the relevance of CAD for short- and long-term outcomes following LuTx and identify risk factors for mortality.

**Methods:**

We retrospectively analyzed all adult patients (≥ 18 years) undergoing primary and isolated LuTx between January 2000 and August 2021 at the LMU University Hospital transplant center. Using 1:1 propensity score matching, 98 corresponding pairs of LuTx patients with and without relevant CAD were identified.

**Results:**

Among 1,003 patients having undergone LuTx, 104 (10.4%) had relevant CAD at baseline. There were no significant differences in in-hospital mortality (8.2% vs. 8.2%, p > 0.999) as well as overall survival (HR 0.90, 95%CI [0.61, 1.32], p = 0.800) between matched CAD and non-CAD patients. Similarly, cardiovascular events such as myocardial infarction (7.1% CAD vs. 2.0% non-CAD, p = 0.170), revascularization by percutaneous coronary intervention (5.1% vs. 1.0%, p = 0.212), and stroke (2.0% vs. 6.1%, p = 0.279), did not differ statistically between both matched groups. 7.1% in the CAD group and 2.0% in the non-CAD group (p = 0.078) died from cardiovascular causes. Cox regression analysis identified age at transplantation (HR 1.02, 95%CI [1.01, 1.04], p < 0.001), elevated bilirubin (HR 1.33, 95%CI [1.15, 1.54], p < 0.001), obstructive lung disease (HR 1.43, 95%CI [1.01, 2.02], p = 0.041), decreased forced vital capacity (HR 0.99, 95%CI [0.99, 1.00], p = 0.042), necessity of reoperation (HR 3.51, 95%CI [2.97, 4.14], p < 0.001) and early transplantation time (HR 0.97, 95%CI [0.95, 0.99], p = 0.001) as risk factors for all-cause mortality, but not relevant CAD (HR 0.96, 95%CI [0.71, 1.29], p = 0.788). Double lung transplant was associated with lower all-cause mortality (HR 0.65, 95%CI [0.52, 0.80], p < 0.001), but higher in-hospital mortality (OR 2.04, 95%CI [1.04, 4.01], p = 0.039).

**Conclusion:**

In this cohort, relevant CAD was not associated with worse outcomes and should therefore not be considered a contraindication for LuTx. Nonetheless, cardiovascular events in CAD patients highlight the necessity of control of cardiovascular risk factors and a structured cardiac follow-up.

**Supplementary Information:**

The online version contains supplementary material available at 10.1007/s00392-024-02445-y.

## Introduction

Advanced lung disease is associated with severely impaired quality of life and dramatically reduced life expectancy [1–3]. Though well established, lung transplantation as the ultimate therapeutic option is highly limited by the scarcity of donor organs [4, 5]. Therefore, thorough evaluation and candidate selection is pivotal to ensure individual benefit from transplantation as well as optimal resource allocation. In LuTx candidates, cardiovascular co-morbidities are common and a relevant proportion of patients with advanced lung disease is known to suffer from occult coronary artery disease (CAD) [6–10]. End-stage pulmonary fibrosis (PF) as well as chronic obstructive pulmonary disease (COPD), for example, are associated with an increased prevalence of CAD – reported up to 35% [10–13]. This association is still insufficiently understood and only partially explained by shared risk factors such as tobacco use [14]. Additionally, relevant CAD has become more prevalent in patients undergoing LuTx due to the increasing age of recipients. Consequently, coronary angiography is routinely performed in candidates older than 40 years or those with elevated cardiovascular risk in the majority of transplantation centers. However, the interpretation of the results, particularly regarding their clinical significance for LuTx outcomes, remains challenging.

While CAD has historically been considered a relative contraindication for LuTx [15], small retrospective single-center studies suggested that the presence of CAD prior to LuTx might not significantly increase the rate of peri- and postoperative cardiovascular events or mortality and that outcomes are acceptable in selected patients undergoing coronary revascularization prior to or concomitant with LuTx [16, 17]. However, published data regarding obstructive CAD with coronary artery stenosis > 50% or necessity for revascularization and long-term outcomes are sparse and partially inconclusive. Thus, while some studies found similar overall survival in patients with obstructive CAD, others have demonstrated impaired long-term survival [18–22]. Albeit most studies were limited by small sample sizes and short follow-up periods. Furthermore, contradicting data concerning cardiovascular events and death following LuTx further complicates cardiovascular candidate evaluation. For instance, studies investigating LuTx and coronary artery bypass grafting (CABG) showed that CABG prior to LuTx might be associated with increased mortality [23], while simultaneous CABG on the other hand doesn’t seem to affect survival [24]. Currently, the International Society for Heart and Lung Transplantation (ISHLT), as stated in its 2021 consensus document for the selection of LuTx candidates, does not consider CAD as an absolute contraindication but rather a risk factor for impaired outcomes and a potential marker for an unfavorable phenotype [25].

Overall, the available evidence is insufficient to evaluate the relevance of relevant CAD in the context of LuTx. Therefore, our study aimed to better understand the role of relevant CAD in LuTx by focusing on short- and long-term survival, cardiovascular events and death following LuTx in patients with and without relevant CAD as well as independent risk factors for mortality in this patient population.

## Methods

### Study design and patient selection

We retrospectively analyzed all adult patients (≥ 18 years) undergoing primary and isolated LuTx between January 2000 and August 2021 at the LMU University Hospital transplant center. Patients receiving multi-organ transplantation or re-transplantation were excluded. Patient data were extracted from the central clinical database, with subsequent strict data anonymization. Validity and integrity of the clinical research dataset was controlled by two senior physicians and by our statistical team. Standardized definitions for comorbidities and a data dictionary were used. Statistical analysis was prespecified and the statistical analysis plan was written before the data was received by the statistical analysis team. This is the primary analysis of this dataset, which was exclusively collected to investigate the role of relevant CAD in LuTx patients.

### Selection and management of LuTx patients

Patient selection was performed following interdisciplinary discussion in the transplantation conference and in accordance with contemporary ISHLT selection guidelines [15, 25]. As per institution guidelines, CAD was assessed in all potential LuTx candidates older than 40 years and in patients with either angina pectoris or a profound cardiovascular risk (arterial hypertension and dyslipidaemia) by routine invasive coronary angiography. In patients who had already undergone percutaneous coronary intervention (PCI) before evaluation for a transplantation, coronary angiography was repeated at the time of listing. Patients were not excluded from LuTx because of CAD if (1) the coronary system was correctable, if (2) coronary perfusion in non-invasive stress testing was sufficient, and if (3) left ventricular ejection fraction (LVEF) was not severely impaired (LVEF > 35%). If patients were diagnosed with CAD requiring revascularization during pretransplant cardiovascular evaluation, revascularization was performed according to the European Society of Cardiology (ESC) and the European Association for Cardio-Thoracic Surgery (EACTS) guidelines after interdisciplinary determination of the best individual revascularization strategy. Depending on the urgency of LuTx, listing was postponed between 3 and 6 months after stenting because of dual antiplatelet therapy. At the time of listing, P2Y12-inhibitors were stopped with continued single antiplatelet therapy based on aspirin. A center-specific structured surveillance and follow-up including outpatient and inpatient visits was conducted and follow-up schedules were adapted to the requirements of each patient with at least yearly posttransplant follow-ups in stable patients. Standard immunosuppression regimen consisted of a triple combination with steroids, mycophenolate mofetil and tacrolimus.

### Definition of relevant CAD

Relevant CAD was defined as (1) presence of epicardial coronary artery stenosis > 50% seen in the coronary angiography, (2) prior necessity of revascularization by PCI or CABG, or (3) prior myocardial infarction (MI). CAD grading in 1–3 vessel disease was correspondingly defined by presence of either coronary artery stenosis > 50% or prior revascularization in the left anterior descending (LAD), left circumflex artery (LCX) or right coronary artery (RCA). Lesions of the left main artery (LM) were thereby treated as combined lesions of LAD and LCX.

### Clinical endpoints

The primary outcome variable was post-transplant survival, ascertained from the date of transplantation until patient death, date of last follow-up as recorded in the electronic health record or the end of study period. Patients were censored if alive at study termination. Secondary endpoints were obtained from a prospectively recorded data base and included in-hospital mortality, post-transplant cardiovascular (MI, necessity of coronary angiography, PCI or CABG, stroke, thrombosis or pulmonary embolism, atrial fibrillation, and cardiac arrest), non-cardiovascular events (dialysis, reoperation) and cause of death (bleeding, chronic allograft dysfunction, cardiovascular, infection/sepsis, malignancy, multi-organ failure and mors in tabula).

### Risk factor analysis

Based on clinical reasoning and prior evidence from the literature [25–30], we considered the following set of risk factors for in-hospital mortality and all-cause mortality in our study: age at transplantation, sex, hypertension, diabetes, smoking, body mass index (BMI), creatinine, bilirubin, restrictive lung disease, obstructive lung disease, forced expiratory volume in 1 s (FEV1), forced vital capacity (FVC), CAD, LVEF, tricuspid annular plane systolic excursion (TAPSE), tricuspid valve regurgitation (TR), mean pulmonary artery pressure (mPAP), pulmonary capillary wedge pressure (PCWP), double lung transplantation, reoperation, bridge to transplant using extracorporeal membrane oxygenation (ECMO) and time since first transplantation, i.e. time difference between the transplantation time of the individual patient and the first transplantation on January 5th, 2000, to take transplant era into account. We performed risk factor analysis using Cox regression and logistic regression for all-cause mortality and in-hospital mortality, respectively. In order to avoid overfitting, we did not perform variable selection on this set of pre-specified risk factors but included all variables in the multiple logistic regression model.

### Ethics approval

The study was conducted in accordance with the Declaration of Helsinki and was approved by the local ethics boards. Ethical approval for this study was obtained from the university hospital’s institutional ethic committee (approval number Az 19–630).

### Statistical analysis

Statistical analysis was performed using R® (version 4.2.1, The R Foundation, Vienna, Austria). Kolmogorov–Smirnov-test, D’Agostino–Pearson-omnibus-test, q-q-plots and histograms were used to test the normality of data distribution. Normally distributed continuous variables were reported as a mean with standard deviation and non-normally distributed continuous variables as a median with interquartile ranges (25th and 75th percentile). To compare two groups, paired and unpaired t-test for normally distributed continuous variables and paired and unpaired Mann–Whitney-U test for non-normally distributed continuous variables were used. Categorical variables were reported as absolute numbers and percentages. To compare unpaired groups, chi-squared test and for matched paired groups, McNemar’s test was used. All tests were 2-tailed, and p-values < 0.05 were considered significant. Survival probabilities were calculated using the Kaplan–Meier method and comparisons were made by using stratified log-rank tests. For propensity score matching, the R package “MatchIt” (version 4.5.0) was utilized with a 1:1 nearest neighbor algorithm, no replacement, logistic link distance measure and a cutoff threshold of 0.2 standardized mean differences was applied. The propensity score was estimated by logistic regression. Based on previous published literature as well as clinical rationale the following baseline parameters were used for matching: age at time of transplantation, sex, BMI, diabetes, hypertension, smoking, bridge to transplant using ECMO, double lung transplantation, FEV1, FVC, CI, mPAP and PCWP [25–30].

## Results

### Study population

**Fig. 1:**
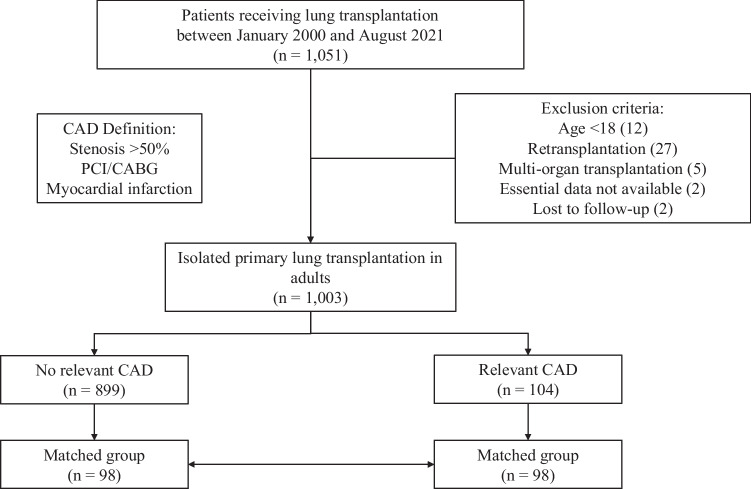
Flow diagram depicting patient selection. 1,051 patients receiving lung transplantation between 01/2000 and 08/2021 were screened, 48 patients met exclusion criteria and were removed from the analysis. A total of 1,003 patients was included and divided in patients with relevant coronary artery disease (n = 104) and patients without relevant coronary artery disease (n = 899). Using 1:1 propensity score matching 98 corresponding pairs of LuTx patients with and without relevant CAD were identified. CAD = coronary artery disease, PCI = Percutaneous coronary intervention, CABG = coronary artery bypass graft

During the study period, a total of 1,051 LuTx patients were evaluated. After exclusion of pediatric patients (12), patients undergoing re-transplantation (27) or multi-organ transplantation (5) and patients with incomplete data or missing follow-up (4), 1,003 patients were included in the analysis (Fig. 1). In total, 98 LuTx patients with relevant CAD could be matched with 98 LuTx patients without CAD, achieving a standard difference of mean below 0.2 for all matching parameters (Supp. Figure 1). The median follow-up was 3.24 [1.30, 4.82] years. In the overall cohort, the median age was 54.7 years [45.8, 60.5] with 547 (54.5%) being male. Restrictive lung disease was the most frequent reason for LuTx (502, 50.0%), followed by obstructive lung disease (289, 28.8%), cystic fibrosis (139, 13.9%), and pulmonary vascular disease (40, 4.0%). The prevalence of CAD including mild CAD and coronary sclerosis was 23%. Relevant CAD was present in 104 (10.4%) patients, with the majority suffering from 1-vessel CAD (64, 6.4%), followed by 2-vessel (23, 2.3%) and 3-vessel disease (17, 1.7%). The most frequently affected coronary artery was the LAD (51, 5.1%), followed by RCA (28, 2.8%), LCX (24, 2.4%) and LM (2, 0.2%). 19 patients (1.9%) had suffered prior MI and revascularization had been performed in 60 patients (6.0%) via PCI while 4 patients (0.4%) had undergone CABG. Detailed baseline characteristics before and after matching can be found in Table 1 and 2, respectively.

**Table 1:** Baseline characteristics of unmatched LuTx patients without and with relevant CAD

Baseline characteristics of unmatched LuTx patients without and with relevant CAD				
Characteristics	Overall (n = 1003)	Patients without CAD (n = 899)	Patients with CAD (n = 104)	p-value
Demographics				
Age [years], median [IQR]	54.73 [45.78, 60.53]	53.96 [44.45, 59.85]	60.28 [56.71, 63.24]	< 0.001
Sex [male], n (%)	547 (54.5)	470 (52.3)	77 (74.0)	< 0.001
Body mass index [kg/m^2^], median [IQR]	22.39 [19.52, 25.90]	22.22 [19.37, 25.62]	24.13 [21.25, 27.41]	< 0.001
Diagnosis				
Restrictive lung disease, n (%)	502 (50.0)	428 (47.6)	74 ( 71.2)	< 0.001
Obstructive lung disease, n (%)	289 (28.8)	259 (28.8)	30 ( 28.8)	> 0.999
Vascular lung disease, n (%)	40 (4.0)	40 ( 4.4)	0 ( 0.0)	0.017
Cystic fibrosis, n (%)	139 (13.9)	139 (15.5)	0 ( 0.0)	< 0.001
Other, n (%)	33 (3.3)	33 ( 3.7)	0 ( 0.0)	0.041
Cardiovascular risk factors				
Hypertension, n (%)	359 (35.8)	289 (32.1)	70 ( 67.3)	< 0.001
Diabetes, n (%)	227 (22.6)	207 (23.0)	20 ( 19.2)	0.458
Smoking, n (%)	438 (43.7)	368 (40.9)	70 ( 67.3)	< 0.001
Cholesterol [mg/dl], median [IQR]	159.00 [124.25, 204.00]	160.00 [124.00, 205.75]	147.00 [126.50, 184.00]	0.146
Morbidity at admission				
Prior Myocardial infarction, n (%)	19 ( 1.9)	0 ( 0.0)	19 ( 18.3)	< 0.001
Prior CABG, n (%)	4 ( 0.4)	0 ( 0.0)	4 ( 3.8)	< 0.001
Prior PCI, n (%)	60 ( 6.0)	0 ( 0.0)	60 ( 57.7)	< 0.001
Prior Stroke, n (%)	19 ( 1.9)	17 ( 1.9)	2 ( 1.9)	> 0.999
Prior Pulmonary embolism, n (%)	67 ( 6.7)	58 ( 6.5)	9 ( 8.7)	0.405
Prior Thrombosis, n (%)	43 ( 4.3)	36 ( 4.0)	7 ( 6.7)	0.200
Prior PVD	15 ( 1.5)	9 ( 1.0)	6 ( 5.8)	0.003
Prior Atrial fibrillation, n (%)	43 ( 4.3)	38 ( 4.2)	5 ( 4.8)	0.800
LTOT, n (%)	996 (99.3)	895 (99.6)	101 ( 97.1)	0.028
CAD Characteristics				
Coronary sclerosis, n (%)	230 (22.9)	126 ( 14.0)	104 (100.0)	< 0.001
1-Vessel-Disease, n (%)	64 ( 6.4)	0 ( 0.0)	64 ( 61.5)	
2- Vessel-Disease, n (%)	23 ( 2.3)	0 ( 0.0)	23 ( 22.1)	
3- Vessel-Disease, n (%)	17 ( 1.7)	0 ( 0.0)	17 ( 16.3)	
Stenosis > 50% LM, n (%)	2 ( 0.2)	0 ( 0.0)	2 ( 1.9)	
Stenosis > 50% LAD, n (%)	51 ( 5.1)	0 ( 0.0)	51 ( 49.0)	
Stenosis > 50% LCX, n (%)	24 ( 2.4)	0 ( 0.0)	24 ( 23.1)	
Stenosis > 50% RCA, n (%)	28 ( 2.8)	0 ( 0.0)	28 ( 26.9)	
Prior PCI LM, n (%)	6 ( 0.6)	0 ( 0.0)	6 ( 5.8)	
Prior PCI LAD, n (%)	30 ( 3.0)	0 ( 0.0)	30 (28.8)	
Prior PCI LCX, n (%)	15 ( 1.5)	0 ( 0.0)	15 (14.4)	
Prior PCI RCA, n (%)	23 ( 2.3)	0 ( 0.0)	23 (22.1)	
Prior CABG, n (%)	4 ( 0.4)	0 ( 0.0)	4 ( 3.8)	
CTO, n (%)	4 ( 0.4)	0 ( 0.0)	4 ( 3.8)	
Hemodynamics				
Cardiac Index [L/m^2^], median [IQR]	3.10 [2.60, 3.60]	3.10 [2.68, 3.70]	2.95 [2.50, 3.40]	0.012
mPAP [mmHg], median [IQR]	25.00 [20.00, 32.00]	25.00 [20.00, 33.00]	24.00 [19.75, 29.00]	0.046
PCWP [mmHg], median [IQR]	9.00 [6.00, 11.00]	9.00 [6.00, 11.00]	8.00 [5.00, 11.00]	0.174
PVR [WE], median [IQR]	2.80 [2.00, 4.43]	2.83 [2.00, 4.50]	2.70 [2.00, 4.01]	0.413
Functional tests				
6MWD [m], median [IQR]	220.00 [100.00, 320.00]	220.00 [100.00, 320.00]	183.00 [90.00, 320.00]	0.357
FVC [% of reference], median [IQR]	40.40 [32.00, 53.00]	40.00 [31.00, 52.70]	43.00 [35.00, 54.70]	0.074
FEV1 [% of reference], median [IQR]	31.00 [21.00, 47.00]	30.00 [20.55, 46.00]	44.00 [23.00, 56.25]	< 0.001
LVEF				
> 55 [%], n (%)	933 (93.0)	842 (93.7)	91 ( 87.5)	0.027
45 – 55 [%], n (%)	48 ( 4.8)	39 ( 4.3)	9 ( 8.7)	0.083
35 – 45 [%], n (%)	13 ( 1.3)	10 ( 1.1)	3 ( 2.9)	0.144
TAPSE				
> 16 [mm], n (%)	909 (90.6)	816 (90.8)	93 ( 89.4)	0.682
< 16 [mm], n (%)	69 ( 6.9)	61 ( 6.8)	8 ( 7.7)	0.682
Valve Disease				
No valve disease > II°, n (%)	855 (85.2)	767 (85.3)	88 ( 84.6)	0.881
Aortic valve stenosis > II°, n (%)	3 ( 0.3)	2 ( 0.2)	1 ( 1.0)	0.280
Aortic valve regurgitation > II°, n (%)	5 ( 0.5)	4 ( 0.4)	1 ( 1.0)	0.422
Mitral valve regurgitation > II°, n (%)	7 ( 0.7)	6 ( 0.7)	1 ( 1.0)	0.536
Tricuspid valve regurgitation > II°, n (%)	121 (12.1)	110 (12.2)	11 ( 10.6)	0.751
Duplex of the A. carotis				
No plaques, n (%)	884 (88.9)	805 (90.4)	79 ( 76.0)	< 0.001
Plaques < 50%, n (%)	97 ( 9.8)	76 ( 8.5)	21 ( 20.2)	< 0.001
Stenosis > 50%, n (%)	13 ( 1.3)	9 ( 1.0)	4 ( 3.8)	0.038
Lab results				
Creatinine [mg/dl], median [IQR]	0.80 [0.70, 1.00]	0.80 [0.70, 1.00]	0.90 [0.80, 1.00]	0.003
Bilirubin [mg/dl], median [IQR]	0.50 [0.30, 0.70]	0.50 [0.30, 0.70]	0.50 [0.30, 0.70]	0.528
Blood Group				
A, n (%)	438 (43.7)	392 (43.6)	46 ( 44.2)	0.917
B, n (%)	161 (16.1)	142 (15.8)	19 ( 18.3)	0.484
AB, n (%)	52 ( 5.2)	51 ( 5.7)	1 ( 1.0)	0.035
0, n (%)	352 (35.1)	314 (34.9)	38 ( 36.5)	0.746
Procedural characteristics				
ECMO bridge to transplant, n (%)	67 ( 6.7)	63 ( 7.0)	4 ( 3.8)	0.299
Double lung transplantation, n (%)	732 (73.0)	684 (76.1)	48 ( 46.2)	< 0.001
Reoperation within hospital stay, n (%)	272 (27.1)	255 (28.4)	17 ( 16.3)	0.010

Baseline characteristics of lung transplant patients with (n = 104) and without (n = 899) relevant coronary artery disease transplanted between 01/2000 and 08/2021 at the LMU University Hospital. CAD = coronary artery disease, CABG = coronary artery bypass graft, PCI = percutaneous coronary intervention, PVD = peripheral vessel disease, LTOT = long term oxygen therapy, LM = left main artery, LAD = left anterior descending artery, LCX = left circumflex artery, RCA = right coronary artery, CTO = chronic total occlusion, mPAP = mean pulmonary artery pressure, PCWP = pulmonary capillary wedge pressure, PVR = pulmonary vascular resistance, 6MWD = six minute walk distance, FVC = forced vital capacity, FEV1 = forced expiratory volume in 1 s, LVEF = left ventricular ejection fraction, TAPSE = tricuspid annular plane systolic excursion, ECMO = extracorporeal membrane oxygenation, IQR, interquartile range. p-values < 0.05 were considered as significant

**Table 2:** Baseline characteristics of matched patients without and with relevant CAD

Baseline characteristics of matched patients without and with relevant CAD				
Characteristics	Patients without CAD (*n* = 98)	Patients with CAD (*n* = 98)	p-value	Absolute SMD
Demographics				
Age [years], median [IQR]	60.00 [57.00, 63.00]	60.00 [56.00, 63.00]		0.077
Sex [male], n (%)	73 (74.5)	71 (72.4)		0.047
Body mass index [kg/m^2^], median [IQR]	23.63[20.61, 27.44]	24.08 [21.22, 27.62]		0.065
Diagnosis				
Restrictive lung disease, n (%)	61 (62.2)	69 (70.4)	0.290	
Obstructive lung disease, n (%)	35 (35.7)	29 (29.6)	0.446	
Vascular lung disease, n (%)	1 (1.0)	0 (0.0)	> 0.999	
Cystic fibrosis, n (%)	0 (0.0)	0 (0.0)	-	
Other, n (%)	1 (1.0)	0 (0.0)	> 0.999	
Cardiovascular risk factors				
Hypertension, n (%)	59 (60.2)	65 (66.3)		0.131
Diabetes, n (%)	18 (18.4)	19 (19.4)		0.026
Smoking, n (%)	70 (71.4)	65 (66.3)		0.109
Cholesterol [mg/dl], median [IQR]	153.00 [125.50, 195.00]	145.00 [125.00, 182.25]	0.333	
Morbidity at admission				
Prior Myocardial infarction, n (%)	0 (0.0)	17 (17.3)	< 0.001	
Prior CABG, n (%)	0 (0.0)	3 (3.1)	0.246	
Prior PCI, n (%)	0 (0.0)	55 (56.1)	< 0.001	
Prior Stroke, n (%)	3 (3.1)	2 (2.0)	> 0.999	
Prior Pulmonary embolism, n (%)	3 (3.1)	8 (8.2)	0.213	
Prior Thrombosis, n (%)	2 (2.0)	7 (7.1)	0.170	
Prior PVD	2 (2.0)	5 (5.1)	0.445	
Prior Atrial fibrillation, n (%)	7 (7.1)	5 (5.1)	0.767	
LTOT, n (%)	98 (100.0)	95 (96.9)	0.246	
CAD Characteristics				
Coronary sclerosis, n (%)	27 (27.6)	98 (100.0)	< 0.001	
1-Vessel-Disease, n (%)	0 (0.0)	62 (63.3)		
2- Vessel-Disease, n (%)	0 (0.0)	21 (21.4)		
3- Vessel-Disease, n (%)	0 (0.0)	15 (15.3)		
Stenosis > 50% LM, n (%)	0 (0.0)	2 (2.0)		
Stenosis > 50% LAD, n (%)	0 (0.0)	48 (49.0)		
Stenosis > 50% LCX, n (%)	0 (0.0)	22 (22.4)		
Stenosis > 50% RCA, n (%)	0 (0.0)	25 (25.5)		
Prior PCI LM, n (%)	0 (0.0)	6 (6.1)		
Prior PCI LAD, n (%)	0 (0.0)	27 (27.6)		
Prior PCI LCX, n (%)	0 (0.0)	15 (15.3)		
Prior PCI RCA, n (%)	0 (0.0)	19 (19.4)		
Prior CABG, n (%)	0 (0.0)	3 (3.1)		
CTO, n (%)	0 (0.0)	4 (4.1)		
Hemodynamics				
Cardiac Index [L/m^2^], median [IQR]	3.00 [2.62, 3.40]	3.00 [2.52, 3.40]		0.095
mPAP [mmHg], median [IQR]	24.00 [19.00, 30.00]	24.00 [20.00, 29.00]		0.148
PCWP [mmHg], median [IQR]	8.00 [6.00, 11.00]	8.00 [5.00, 11.00]		0.011
PVR [WE], median [IQR]	2.70 [1.85, 3.85]	2.80 [2.00, 4.06]	0.562	
Functional tests				
6MWD [m], median [IQR]	240.00 [120.00, 317.50]	181.50 [87.50, 312.50]	0.161	
FVC [% of reference], median [IQR]	43.00 [35.00, 56.00]	43.00 [35.00, 54.45]		0.066
in obstructive lung disease, median [IQR]	45.00 [35.50, 53.50]	39.00 [33.00, 51.00]		
in restrictive lung disease, median [IQR]	39.85 [32.00, 56.00]	43.00 [35.00, 54.00]		
FEV1 [% of reference], median [IQR]	40.00 [22.10, 58.50]	42.00 [23.00, 56.00]		0.077
in obstructive lung disease, median [IQR]	21.00 [19.00, 25.75]	21.00 [20.00, 23.00]		
in restrictive lung disease, median [IQR]	47.50 [36.75, 63.25]	50.00 [40.00, 59.00]		
LVEF				
> 55 [%], n (%)	90 (91.8)	85 (86.7)	0.126	
45 – 55 [%], n (%)	5 (5.1)	9 (9.2)	0.406	
35 – 45 [%], n (%)	0 (0.0)	3 (3.1)	0.246	
TAPSE				
> 16 [mm], n (%)	87 (88.8)	88 (89.8)	> 0.999	
< 16 [mm], n (%)	7 (7.1)	7 (7.1)	> 0.999	
Valve Disease				
No valve disease > II°, n (%)	82 (83.7)	83 (84.7)	> 0.999	
Aortic valve stenosis > II°, n (%)	0 (0.0)	1 (1.0)	> 0.999	
Aortic valve regurgitation > II°, n (%)	1 (1.0)	1 (1.0)	> 0.999	
Mitral valve regurgitation > II°, n (%)	0 (0.0)	1 (1.0)	> 0.999	
Tricuspid valve regurgitation > II°, n (%)	11 (11.2)	11 (11.2)	> 0.999	
Duplex of the A. carotis				
No plaques, n (%)	83 (84.7)	76 (77.6)	0.135	
Plaques < 50%, n (%)	11 (11.2)	18 (18.4)	0.227	
Stenosis > 50%, n (%)	2 (2.0)	4 (4.1)	0.683	
Lab results				
Creatinine [mg/dl], median [IQR]	0.90 [0.80, 1.08]	0.90 [0.80, 1.00]	0.860	
Bilirubin [mg/dl], median [IQR]	0.50 [0.30, 0.70]	0.50 [0.32, 0.70]	0.403	
Blood Group				
A, n (%)	46 (46.9)	43 (43.9)	0.774	
B, n (%)	16 (16.3)	18 (18.4)	0.851	
AB, n (%)	6 (6.1)	1 (1.0)	0.118	
0, n (%)	30 (30.6)	36 (36.7)	0.450	
Procedural characteristics				
ECMO bridge to transplant, n (%)	3 (3.1)	4 (4.1)		0.053
Double lung transplantation, n (%)	46 (46.9)	48 (49.0)		0.041
Reoperation within hospital stay, n (%)	16 (16.3)	16 (16.3)	> 0.999	

Comparison of 1:1 propensity score matched lung transplant patients with (n = 98) and without (n = 98) relevant coronary artery disease. CAD = coronary artery disease, CABG = coronary artery bypass graft, PCI = percutaneous coronary intervention, PVD = peripheral vessel disease, LTOT = long term oxygen therapy, LM = left main artery, LAD = left anterior descending artery, LCX = left circumflex artery, RCA = right coronary artery, CTO = chronic total occlusion, mPAP = mean pulmonary artery pressure, PCWP = pulmonary capillary wedge pressure, PVR = pulmonary vascular resistance, 6MWD = six minute walk distance, FVC = forced vital capacity, FEV1 = forced expiratory volume in 1 s, LVEF = left ventricular ejection fraction, TAPSE = tricuspid annular plane systolic excursion, ECMO = extracorporeal membrane oxygenation, IQR, interquartile range, p-values < 0.05 were considered as significant; SMD, Standardized Mean Difference. p-values < 0.05 were considered as significant

### Differences in baseline characteristics between patients without and with relevant CAD

Clinical and practical significant differences between unmatched patients with and without relevant CAD were observed concerning age at transplantation [y] (60.3 [56.7, 63.2] vs. 54.0 [44.5, 59.9], p < 0.001), sex [male] (74.0% vs. 52.3%, p < 0.001) and BMI [kg/m^2^] (24.1 [21.3, 27.4] vs. 22.2 [19.4, 25.6], p < 0.001) as well as the abundance of cardiovascular risk factors including hypertension (67.3% vs. 32.1%, p < 0.001) and history of tobacco use (67.3% vs. 40.9%, p < 0.001). Furthermore, patients with relevant CAD underwent transplants more often for restrictive lung disease (71.2% vs. 47.6%, p < 0.001), had lower CI [L/m^2^] (2.95 [2.50, 3.40] vs. 3.10 [2.68, 3.70], p = 0.012), more often reduced LVEF (11.6% vs. 5.4%, p = 0.027), a higher rate of carotid plaques (20.2% vs. 8.5%, p < 0.001) and a higher FEV1 [% of reference] (44.00 [23.00, 56.25] vs. 30.00 [20.55, 46.00], p < 0.001) (Tab. 1). In the matched cohort, patients with relevant CAD significantly more often had coronary sclerosis (100% vs. 27.6%, p < 0.001), suffered prior MI (17.3% vs. 0.0%, p < 0.001) and underwent prior PCI (56.1% vs. 0.0%, p < 0.001) (Table 2).

### Survival

**Fig. 2:**
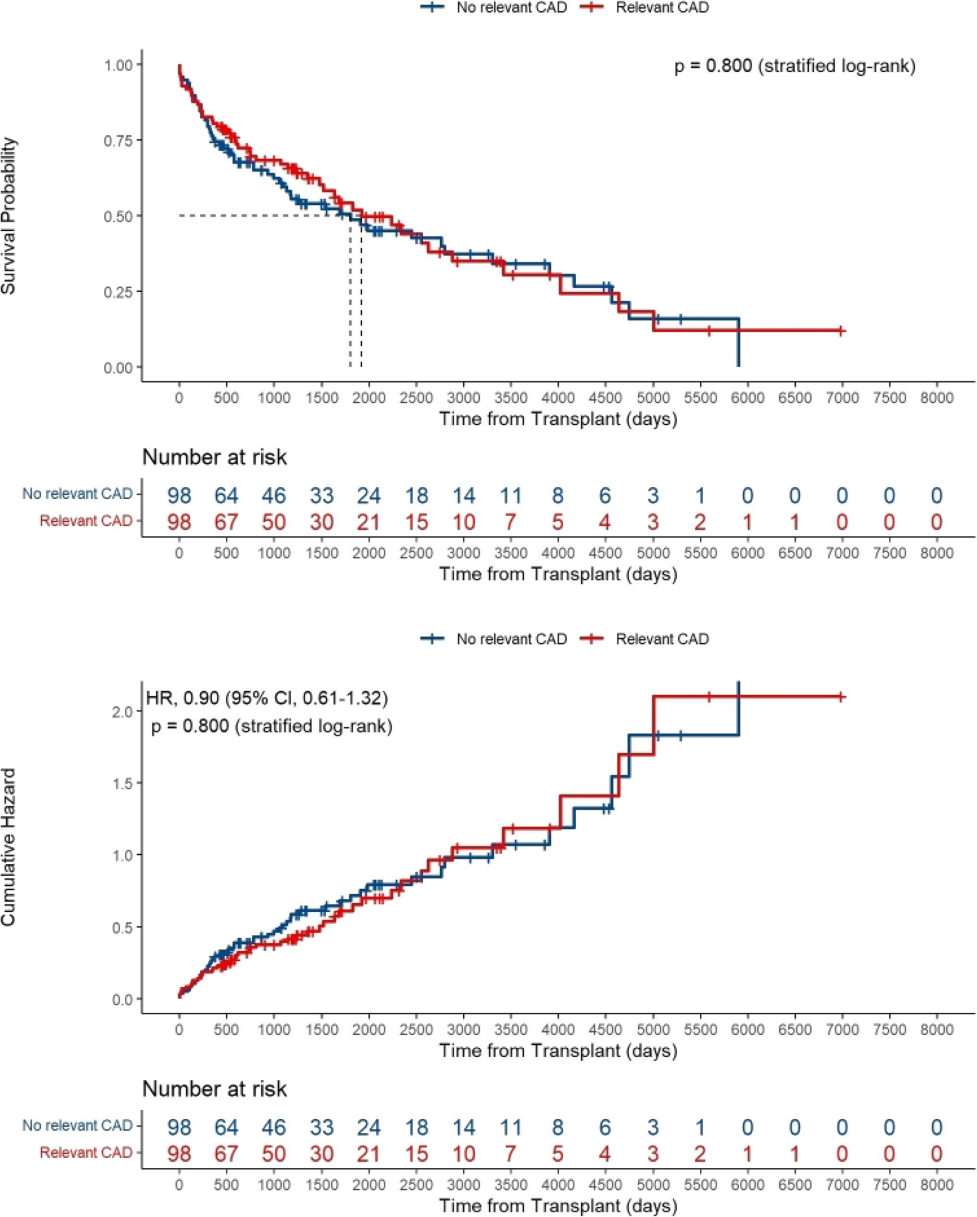
Kaplan–Meier analysis of propensity score matched lung transplant patients with vs. without relevant coronary artery disease. CAD = coronary artery disease, HR = hazard ration, CI = confidence interval

Survival was not significantly different between the two matched groups (HR = 0.90, 95% CI 0.61–1.32, p = 0.800) as shown in Fig. 2 (corresponding Kaplan–Meier analysis for the unmatched cohort (HR = 1.23, 95% CI 0.92–1.64, p = 0.167) is shown in Supp. Figure 2, and for no CAD vs. (I) 1- or 2-vessel disease, vs. (II) 3-vessel disease, vs. (III) prior MI, and vs. (IV) prior revascularization in Supp. Figure 3–6, respectively, accordingly without significant differences in survival). In addition, no significant difference in in-hospital mortality between LuTx patients with and without relevant CAD (8.2% vs. 8.2%, p > 0.999) could be detected (Table 3) (corresponding in-hospital analysis for the unmatched cohort is shown in Supp. Tab. 1–2).

**Table 3:** In-hospital mortality in matched patients without and with relevant CAD

In-hospital mortality in matched patients without and with relevant CAD			
Characteristics	Patients without CAD (*n* = 98)	Patients with CAD (*n* = 98)	*p*-value
Survival			
In-hospital mortality	8 (8.2)	8 (8.2)	> 0.999

In-hospital mortality in matched patients without and with CAD. CAD = coronary artery disease. p-values < 0.05 were considered as significant

### Cardiovascular events following LuTx

Coronary angiography was required significantly more often in the matched CAD group (17.3% CAD vs. 5.1% non-CAD, p = 0.011). However, MI (7.1% CAD vs. 2.0% non-CAD, p = 0.170), PCI (5.1% vs. 1.0%, p = 0.212) and CABG (1% vs. 0%, p > 0.999) occurred numerically more frequently in the CAD group, but without reaching statistical significance. Stroke (2.0% vs. 6.1%, p = 0.279), atrial fibrillation (new onset) (15.3% vs. 23.5%, p = 0.206), dialysis (8.2% vs. 11.2%, p = 0.630) as well as cardiac arrest (6.1% vs. 11.2%, p = 0.310) were more frequent in the non-CAD group (Table 4, unmatched cohort is shown in Supp. Tab. 3–4) albeit without reaching statistical significance.

**Table 4:** Adverse events in matched patients without and with relevant CAD

Adverse events in matched patients without and with relevant CAD			
Characteristics	Patients without CAD (*n* = 98)	Patients with CAD (*n* = 98)	p-value
Adverse Events post transplantation			
Myocardial Infarction, n (%)	2 (2.0)	7 (7.1)	0.170
Coronary angiography, n (%)	5 (5.1)	17 (17.3)	0.011
PCI, n (%)	1 (1.0)	5 (5.1)	0.212
CABG, n (%)	0 (0.0)	1 (1.0)	> 0.999
Stroke, n (%)	6 (6.1)	2 (2.0)	0.279
Pulmonary embolism, n (%)	11 (11.2)	14 (14.3)	0.669
Thrombosis, n (%)	18 (18.4)	16 (16.3)	0.851
Cardiac arrest, n (%)	11 (11.2)	6 (6.1)	0.310
Atrial fibrillation (new onset), n (%)	23 (23.5)	15 (15.3)	0.206
Dialysis, n (%)	11 (11.2)	8 (8.2)	0.630
Re-Operation, n (%)	16 (16.3)	16 (16.3)	> 0.999

Comparison of cardiovascular events in 1:1 propensity score matched patients with and without relevant coronary artery disease following lung transplantation. CAD = coronary artery disease, PCI = percutaneous coronary intervention, CABG = coronary artery bypass graft. p-values < 0.05 were considered as significant

### Cause of death following LuTx

Sepsis and infectious disease were the most common cause of death in LuTx patients (15.3% CAD vs. 16.3% non-CAD, p = 0.832), followed by chronic allograft dysfunction (11.2% vs. 6.1%, p = 0.118). Cardiovascular death occurred more often in the CAD group (7.1% vs. 2.0%, p = 0.078), albeit not reaching statistical significance. Further details on cause of death can be found in Table 5 (unmatched cohort is shown in Supp. Tab. 5–6).

**Table 5:** Cause of Death in matched patients without and with relevant CAD

Cause of Death in matched patients without and with relevant CAD			
Characteristics	Patients without CAD (*n* = 98)	Patients with CAD (*n* = 98)	p-value
Cause of Death			
Bleeding, n (%)	6 (6.1)	2 (2.0)	0.279
Chronic lung allograft dysfunction, n (%)	6 (6.1)	11 (11.2)	0.118
Cardiovascular, n (%)	2 (2.0)	7 (7.1)	0.078
Infection/Sepsis, n (%)	16 (16.3)	15 (15.3)	0.832
Malignancy, n (%)	4 (4.1)	4 (4.1)	> 0.999
Multi-organ failure, n (%)	7 (7.1)	4 (4.1)	0.537
Mors in tabula, n (%)	1 (1.0)	1 (1.0)	> 0.999
Other, n (%)	2 (2.0)	2 (2.0)	> 0.999
Unknown, n (%)	11 (11.2)	2 (2.0)	0.018

Cause of death in 1:1 propensity score matched patients with and without relevant coronary artery disease following lung transplantation. CAD = coronary artery disease. p-values < 0.05 were considered as significant

### Risk factors for in-hospital mortality in LuTx patients

The logistic regressions models revealed the following set of independent risk factors for in-hospital mortality: age at transplantation [years] (Odds Ratio [OR] 1.04, 95% confidence interval [CI] [1.01, 1.07], p = 0.009), elevated bilirubin [mg/dl] (OR 1.90, 95%CI [1.19, 3.03], p = 0.007), decreased forced vital capacity [% of reference] (OR 0.97, 95%CI [0.95, 0.99], p = 0.004), double lung transplantation (OR 2.04, 95%CI [1.04, 4.01], p = 0.039) and necessity of reoperation (OR 2.99, 95%CI [1.84, 4.87], p < 0.001) were associated with higher in-hospital mortality. Time since first transplantation [years] (OR 0.93, 95%CI [0.89, 0.97], p = 0.001) was associated with lower in-hospital mortality. However, relevant CAD (OR 1.30, 95%CI [0.55, 3.09], p = 0.547) did not emerge as a risk factor for in-hospital mortality. The results are summarized in Table 6.

**Table 6:** Risk factors for (A) in-hospital mortality and (B) all-cause mortality

(A) Risk factors for in-hospital mortality				
Attribute	Univariate analysis		Multivariate analysis	
	OR [95% CI]	*p*-value	OR [95% CI]	*p*-value
Age at Tx [years]	0.99 [0.98, 1.01]	0.570	1.04 [1.01, 1.07]	0.009
Sex [male]	1.18 [0.76, 1.83]	0.457	1.15 [0.70, 1.89]	0.582
Hypertension [yes]	1.02 [0.65, 1.60]	0.922	1.13 [0.68, 1.89]	0.640
Diabetes [yes]	0.96 [0.57, 1.61]	0.876	0.98 [0.53, 1.79]	0.937
Smoking [yes]	0.71 [0.46, 1.11]	0.137	0.82 [0.45, 1.50]	0.519
Body mass index [kg/${{\text{m}}}^{2}$]	0.99 [0.94, 1.04]	0.613	0.96 [0.90, 1.03]	0.277
Kreatinin [mg/dl]	1.36 [0.69, 2.69]	0.378	1.08 [0.48, 2.42]	0.855
Bilirubin [mg/dl]	2.21 [1.49, 3.29]	< 0.001	1.90 [1.19, 3.03]	0.007
Restrictive lung disease [yes]	0.80 [0.52, 1.24]	0.319	0.47 [0.21, 1.05]	0.066
Obstructive lung disease [yes]	0.87 [0.54, 1.43]	0.590	0.78 [0.31, 2.01]	0.612
FEV1 [% of reference]	1.00 [0.99, 1.01]	0.902	1.00 [0.98, 1.03]	0.667
FVC [% of reference]	0.99 [0.98, 1.01]	0.269	0.97 [0.95, 0.99]	0.004
Relevant CAD [yes]	0.82 [0.38, 1.75]	0.605	1.30 [0.55, 3.09]	0.547
LVEF [abnormal]	3.34 [1.17, 4.68]	0.016	1.94 [0.87, 4.33]	0.106
TAPSE [reduced]	2.81 [1.50, 5.30]	0.001	1.95 [0.86, 4.41]	0.108
TI [$>$ °II]	3.08 [1.29, 7.38]	0.011	1.93 [0.64, 5.82]	0.245
mPAP [mmHg]	1.03 [1.01, 1.04]	0.001	1.02 [0.99, 1.04]	0.179
PCWP [mmHg]	1.05 [1.00, 1.10]	0.046	1.03 [0.98, 1.09]	0.287
Double Lung Transplant [yes]	1.82 [1.04, 3.18]	0.036	2.04 [1.04, 4.01]	0.039
Reoperation [yes]	3.26 [2.10, 5.06]	< 0.001	2.99 [1.84, 4.87]	< 0.001
ECMO [yes]	1.85 [0.91, 3.77]	0.089	1.40 [0.56, 3.53]	0.475
Time since first transplantation at all in the study [years]	0.96 [0.93, 1.00]	0.036	0.93 [0.89, 0.97]	0.001
(B) Risk factors for all-cause mortality				
Attribute	Univariate analysis		Multivariate analysis	
	HR [95% CI]	p-value	HR [95% CI]	p-value
Age at Tx [years]	1.03 [1.02, 1.04]	< 0.001	1.02 [1.01, 1.04]	< 0.001
Sex [male]	1.27 [1.06, 1.52]	0.009	1.13 [0.95, 1.34]	0.159
Hypertension [yes]	1.25 [1.05, 1.50]	0.015	0.98 [0.83, 1.16]	0.831
Diabetes [yes]	0.93 [0.75, 1.15]	0.475	1.15 [0.94, 1.40]	0.178
Smoking [yes]	1.26 [1.06, 1.51]	0.011	0.88 [0.71, 1.08]	0.217
Body mass index [kg/${{\text{m}}}^{2}$]	1.04 [1.01, 1.06]	0.001	0.99 [0.97, 1.02]	0.622
Kreatinin [mg/dl]	1.55 [1.16, 2.07]	0.003	1.21 [0.88, 1.66]	0.233
Bilirubin [mg/dl]	1.33 [1.11, 1.59]	0.002	1.33 [1.15, 1.54]	< 0.001
Restrictive lung disease [yes]	1.02 [0.85, 1.21]	0.853	1.17 [0.87, 1.57]	0.311
Obstructive lung disease [yes]	1.46 [1.22, 1.76]	< 0.001	1.43 [1.01, 2.02]	0.041
FEV1 [% of reference]	1.00 [0.99, 1.00]	0.543	1.00 [0.99, 1.01]	0.780
FVC [% of reference]	1.00 [0.99, 1.00]	0.769	0.99 [0.99, 1.00]	0.042
Relevant CAD [yes]	1.23 [0.92, 1.64]	0.167	0.96 [0.71, 1.29]	0.788
LVEF [abnormal]	1.40 [0.97, 2.02]	0.072	1.13 [0.82, 1.55]	0.466
TAPSE [reduced]	1.24 [0.88, 1.76]	0.225	1.16 [0.85, 1.59]	0.366
TI [$>$°II]	1.33 [0.78, 2.27]	0.291	1.44 [0.89, 2.35]	0.141
mPAP [mmHg]	1.00 [0.99, 1.01]	0.751	1.01 [1.00, 1.02]	0.108
PCWP [mmHg]	1.03 [1.01, 1.05]	0.004	1.01 [0.99, 1.03]	0.248
Double Lung Transplant [yes]	0.51 [0.43, 0.62]	< 0.001	0.65 [0.52, 0.80]	< 0.001
Reoperation [yes]	2.91 [2.49, 3.39]	< 0.001	3.51 [2.97, 4.14]	< 0.001
ECMO [yes]	0.75 [0.48, 1.18]	0.215	0.94 [0.62, 1.42]	0.758
Time since first transplantation at all in the study [years]	0.98 [0.97, 1.00]	0.068	0.97 [0.95, 0.99]	0.001

Risk factors for (A) in-hospital mortality and (B) all-cause mortality in lung transplant patients. OR = Odds ratio, HR = Hazard ratio, CI = Confidence interval, FVC = forced vital capacity, FEV1 = forced expiratory volume in 1 s, ECMO = Extracorporeal membrane oxygenation, TI = tricuspid valve insufficiency, LVEF = left ventricular ejection fraction, TAPSE = tricuspid annular plane systolic excursion, mPAP = mean pulmonary artery pressure, PCWP = pulmonary capillary wedge pressure, CAD = Coronary artery disease. p-values < 0.05 were considered as significant

### Risk factors for all-cause mortality in LuTx patients

The following set of risk factors were independently related to all-cause mortality: Age at transplantation [years] (Hazard Ratio [HR] 1.02, 95%CI [1.01, 1.04], p < 0.001), elevated bilirubin [mg/dl] (HR 1.33, 95%CI [1.15, 1.54], p < 0.001), obstructive lung disease (HR 1.43, 95%CI [1.01, 2.02], p = 0.041), decreased forced vital capacity [% of reference] (HR 0.99, 95%CI [0.99, 1.00], p = 0.042) and necessity of reoperation (HR 3.51, 95%CI [2.97, 4.14], p < 0.001) were associated with higher all-cause mortality, while time since first transplantation [years] (HR 0.97, 95%CI [0.95, 0.99], p = 0.001) and double lung transplantation (HR 0.65, 95%CI [0.52, 0.80], p < 0.001) were associated with lower all-cause mortality. Similar to in-hospital mortality, relevant CAD (HR 0.96, 95%CI [0.71, 1.29], p = 0.788) was not associated with all-cause mortality. The results are summarized in Table 6.

## Discussion

Even after control for multiple confounders, relevant CAD in lung transplant recipients was not associated with increased mortality in the current analysis. Considering various confounders by propensity score matching and Cox regression analysis, no influence on in-hospital and all-cause mortality was found. Cardiovascular events after transplantation were common, endorsing a rigorous management of cardiovascular risk factors and continuous cardiovascular monitoring as part of the structured follow-up of lung transplant recipients.

The 2021 ISHLT consensus document for the selection of lung transplant candidates lists mild to moderate CAD and revascularized CAD as a risk factor for an unfavourable outcome, but states that CAD should not be considered an absolute contraindication [25]. The current data do not provide evidence for an increased risk of mortality in a highly selected cohort with reduced long-term survival and with other predictors of outcome. Thus, patients with relevant CAD should not be per se excluded from lung transplantation but should rather thorough assessment of comorbidities. It is likely that patients with CAD have been otherwise deemed as good transplant candidates, which must be considered when interpreting our results.

Among 1,003 patients having undergone LuTx, 10.4% had relevant CAD in the present study. This is in line with previous investigations proving a relevant incidence of CAD in LuTx candidates [16–21]. This should not seem surprising since chronic lung disease and CAD share common risk factors, such as advanced age and smoking history. Furthermore, chronic respiratory disease itself is a risk factor for CAD most likely due to chronic inflammation and systemic hypoxia [31, 32]. Thus, the higher cardiovascular risk burden of LuTx candidates and the high incidence of CAD underlines the necessity of preoperative cardiovascular evaluation. In this context, coronary angiography is recommended since non-invasive tests are frequently limited by the advanced stages of lung disease [33, 34].

Overall, our findings contribute to an increasing body of literature studying the impact of CAD on outcomes after LuTx: Castleberry et al. analyzed 791 LuTx recipients between 1997 and 2010 and found no impact of revascularized CAD on risk for death. Preoperative PCI seemed to be superior to concurrent CABG in the context of LuTx [35]. Similarly, Kanaparthi et al. retrospectively investigated 468 patients undergoing single and double lung transplantation and found that preoperative (PCI n = 34, CABG n = 25) or intraoperative revascularization (CABG n = 29) did not negatively impact survival in LuTx patients [36]. In our analysis, preoperative and concurrent CABG were hardly performed, indicating a predominant revascularization strategy by PCI increasing statistical power and facilitating interpretation of results.

In line with our results previous, smaller cohort studies have not demonstrated survival differences between patients with no/mild or relevant CAD, keeping in mind different classifications and observation times [17, 19, 21, 24, 37]. One strength of the current analysis is the large number of analyzed patients as well as respective event rates allowing us to control for several confounders. We performed a detailed assessment in this large propensity-matched cohort, allowing an analysis of the impact of comorbidities and cardiovascular risk factors on outcomes.

Despite no difference in all-cause mortality, it remains unclear whether the presence of CAD is associated with cardiovascular events and cardiovascular deaths following LuTx. Chaikriangkrai et al. reported that relevant CAD was an independent predictor of cardiovascular events in their unmatched analysis (HR 20.32, 95% CI [5.79, 71.26], p < 0.001) concluding that CAD patients are at higher risk of non-fatal cardiovascular events [19]. Furthermore, Zanotti et al. showed that patients with mild-to-moderate CAD needed coronary revascularization more frequently than those without CAD [17]. Similarly, cardiovascular events after transplantation were more common and there was a trend towards more cardiovascular deaths in our cohort. However, significant differences between patients with and without CAD were present, making causality difficult to assess. After matching, differences in cardiovascular events differed without reaching statistical significance. Therefore, CAD may be considered a surrogate for cardiovascular outcome but may not be a risk factor for mortality in the context of a limited overall long-term survival of a lung transplant recipient. In this respect, vigorous and continuous cardiovascular follow-up in LuTx patients with CAD seems indicated. Furthermore, management of cardiovascular risk factors is of particular importance, as immunosuppressive drugs are associated with aggravation of hyperlipidaemia, arterial hypertension and diabetes mellitus [38].

In LuTx the effect of CAD on outcome may be limited by the reduced long-term survival and may be influenced by other outcome defining factors. Age at transplantation, elevated bilirubin, obstructive lung disease, decreased forced vital capacity, necessity of reoperation and early transplantation time were independent risk factors for all-cause mortality, while relevant CAD was not. Although age at transplantation is an anticipated risk factor, the overall survival disadvantage, despite superior in-hospital outcome, of patients with COPD may be explained by additional disease-specific morbidities and is in line with the recent ISHLT report [39]. Preoperative bilirubin can be interpreted as a surrogate parameter for right heart failure in the context of severe pulmonary hypertension. Patients with pulmonary arterial hypertension are at relevant risk for worse outcomes, particularly in the early post-transplantation period, this may be due to the complexities of lung transplantation and postoperative dysfunction of the left ventricle [40]. Furthermore, patients bridged to transplantation via ECMO are at risk for liver injury, as this complication is frequently irreversible and associated with high mortality [41]. While double lung transplantation was a significant risk factor for in-hospital mortality, most likely due to increased operative complexity, it was associated with reduced all-cause mortality. This highlights the long-term benefit of double lung transplantation irrespective of the higher perioperative complexity. However, the survival advantage is driven by a strong selection bias for double lung transplantation of patients deemed in a better overall condition.

This study analysed the outcome of LuTx patients over a study period of more than 20 years. During this time, management of LuTx recipients has advanced. For instance, the introduction of azithromycin therapy for the prevention and treatment of chronic lung allograft dysfunction and the implementation of the Lung Allocation Score in 2011 were significant changes. Finally, center experience contributes to improved outcomes and has evolved over time [42]. Consequently, it does not seem surprising, that in our Cox regression analysis a negative association between time since first transplantation and all-cause mortality was found. Furthermore, time since first transplantation was also negatively associated with in-hospital mortality, indicating improvements in candidate selection, perioperative management and postoperative care. With further improvements and increasing long-term survival after lung transplantation, the association between CAD with cardiovascular events and all-cause mortality may have to be reassessed.

As a final remark, the influence of organ transplantation on CAD is not sufficiently understood. On the one hand, the presence of a continuous inflammation due to alloimmunity has the potential to accelerate atherosclerosis and thereby CAD. On the other hand, the continuous immunosuppressive therapy is thought to decrease the progress of CAD. A clinical study investigating the rate of CAD progression in patients following solid organ transplantation compared to the overall population is direly needed to shed light on this question.

These results should be interpreted within their given limitations. Due to the inherent limitations such as a lack of randomization and blinding to outcomes, our study may be limited by unmeasured confounders and selection bias. We cannot rule out that a longer follow-up period may have led to significant differences in survival. With improvement of long-term outcome after transplantation due to advances in management of immunosuppression and chronic rejection, cardiovascular events might play a more important role in the future. Furthermore, we cannot rule out that some younger patients might have suffered from undetected CAD. However, due to the comprehensive cardiovascular assessment associated with transplant evaluation, this is very unlikely. To avoid overfitting not all potential risk factors for mortality could be included in the regression analysis. However, in contrast to previous studies many confounders were taken into account. Furthermore, due to the very small number of patients receiving CABG, a direct comparison with PCI could not be performed. On the other side the consistent use of PCI as the primary revascularization strategy facilitates interpretation of our results. In addition, patients excluded from LuTx due to cardiovascular evaluation had not been tracked in our system and are therefore unavailable for analysis. In this respect, it is likely that patients with CAD who were selected for transplantation were otherwise regarded as particularly good candidates potentially further affecting outcome analysis.

## Conclusions

CAD is a frequent comorbidity in patients with end-stage lung disease requiring lung transplantation. In the present study, relevant CAD was not associated with short- and long-term survival. Cardiovascular events were more common in patients with CAD, likely due to due to differences in risk factor burden. After adjusting for confounders, no association of CAD and cardiovascular morbidity and mortality was found, indicating the need for rigorous management of cardiovascular risk factors after transplantation. The association of CAD and outcome after lung transplantation may need to be reassessed with improvement of management of transplant outcome defining factors and long-term survival, respectively.

## Supplementary Information

Below is the link to the electronic supplementary material.

Supplementary file1 (DOCX 31 KB)

## Data Availability

The data presented in this study are available on request from the corresponding author. The data are not publicly available due to ethical restrictions and legal constraints.
