# Supplementary material for: The role of coronary artery disease in lung transplantation: a propensity-matched analysis
Source: Clin Res Cardiol. 2024 Apr 8;113(12):1717–32. doi: 10.1007/s00392-024-02445-y (PMC11579179; doi:10.1007/s00392-024-02445-y)
Supplement: Supplementary file 1 — Supplementary file1 (DOCX 31 KB) [file 392_2024_2445_MOESM1_ESM.docx]

**- Supplementary Tables -**

| **In hospital mortality in unmatched patients without and with relevant CAD** | | | | |
| --- | --- | --- | --- | --- |
| **Characteristics** | **Overall (n = 1003)** | **Patients without  CAD**  **(n =899)** | **Patients with CAD**  **(n =104)** | **p-value** |
| **Survival** | | | | |
| In-hospital mortality | 91 (9.1) | 83 (9.2) | 8 (7.7) | 0.720 |

**Supplementary Table 1:** In hospital mortality in unmatched patients without and with relevant CAD. CAD = coronary artery disease. CAD = coronary artery disease. p-values < 0.05 were considered as significant.

| **In-hospital mortality in unmatched patients** | | | | | | | |  |
| --- | --- | --- | --- | --- | --- | --- | --- | --- |
| **Characteristics** | **Overall (n = 1003)** | **Patients without  CAD**  **(n =899)** | **Patients with coronary sclerosis**  **(n = 230)** | **Patients with one or two vessel disease**  **(n = 87)** | **Patients with three vessel disease**  **(n = 17)** | **Patients with myocardial infarction**  **(n = 19)** | **Patients with revascularization**  **(n = 46)** |  |
| **Survival** | | | | | | | | |
| In hospital mortality | 91 (9.1) | 83 (9.2) | 22 (9.6) | 7 (8.0) | 1 (5.9) | 0 (0.0) | 3 (6.5) |  |

**Supplementary Table 2:** In-hospital mortality in unmatched patients. CAD = coronary artery disease.

| **Adverse events in unmatched patients without and with relevant CAD** | | | | |
| --- | --- | --- | --- | --- |
| **Characteristics** | **Overall (n = 1003)** | **Patients without  CAD**  **(n =899)** | **Patients with CAD**  **(n =104)** | **p-value** |
| **Adverse Events post transplantation** | | | | |
| Myocardial Infarction, n (%) | 19 (1.9) | 12 (1.3) | 7 (6.7) | 0.002 |
| Coronary angiography, n (%) | 76 (7.6) | 55 (6.1) | 21 (20.2) | <0.001 |
| PCI, n (%) | 20 (2.0) | 12 (1.3) | 8 (7.7) | <0.001 |
| CABG, n (%) | 1 (0.1) | 0 (0.0) | 1 (1.0) | 0.104 |
| Stroke, n (%) | 44 (4.4) | 42 (4.7) | 2 (1.9) | 0.308 |
| Pulmonary embolism, n (%) | 115 (11.5) | 100 (11.1) | 15 (14.4) | 0.329 |
| Thrombosis, n (%) | 172 (17.1) | 154 (17.1) | 18 (17.3) | >0.999 |
| Cardiac arrest, n (%) | 64 (6.4) | 58 (6.5) | 6 (5.8) | >0.999 |
| Atrial fibrillation (new onset), n (%) | 216 (21.5) | 201 (22.4) | 15 (14.4) | 0.078 |
| Dialysis, n (%) | 111 (11.1) | 103 (11.5) | 8 (7.7) | 0.321 |
| Re-Operation, n (%) | 272 (27.1) | 255 (28.4) | 17 (16.3) | 0.010 |

**Supplementary Table 3:** Adverse events in unmatched patients without and with relevant CAD. CAD = coronary artery disease. CAD = coronary artery disease, PCI = percutaneous coronary intervention, CABG = coronary artery bypass graft. p-values < 0.05 were considered as significant.

| **Adverse events in unmatched patients** | | | | | | | |
| --- | --- | --- | --- | --- | --- | --- | --- |
| **Characteristics** | **Overall (n = 1003)** | **Patients without  CAD**  **(n = 899)** | **Patients with coronary sclerosis**  **(n = 230)** | **Patients with one or two vessel disease**  **(n = 87)** | **Patients with three vessel disease**  **(n = 17)** | **Patients with prior myocardial infarction**  **(n = 19)** | **Patients with prior revascularization**  **(n = 46)** |
| **Adverse Events post transplantation** | | | | | | | |
| Myocardial Infarction, n (%) | 19 (1.9) | 12 (1.3) | 11 (4.8) | 7 (8.0) | 0 (0.0) | 1 (5.3) | 2 (4.3) |
| Coronary angiography, n (%) | 76 (7.6) | 55 (6.1) | 31 (13.5) | 17 (19.5) | 4 (23.5) | 5 (26.3) | 8 (17.4) |
| PCI, n (%) | 20 (2.0) | 12 (1.3) | 10 (4.3) | 6 (6.9) | 2 (11.8) | 2 (10.5) | 3 (6.5) |
| CABG, n (%) | 1 (0.1) | 0 (0.0) | 1 (0.4) | 1 (1.1) | 0 (0.0) | 0 (0.0) | 1 (2.2) |
| Stroke, n (%) | 44 (4.4) | 42 (4.7) | 11 (4.8) | 2 (2.3) | 0 (0.0) | 0 (0.0) | 1 (2.2) |
| Pulmonary embolism, n (%) | 115 (11.5) | 100 (11.1) | 27 (11.7) | 15 (17.2) | 0 (0.0) | 4 (21.1) | 7 (15.2) |
| Thrombosis, n (%) | 172 (17.1) | 154 (17.1) | 42 (18.3) | 16 (18.4) | 2 (11.8) | 8 (42.1) | 10 (21.7) |
| Cardiac arrest, n (%) | 64 (6.4) | 58 (6.5) | 16 (7.0) | 5 (5.7) | 1 (5.9) | 0 (0.0) | 4 (8.7) |
| Atrial fibrillation (new onset), n (%) | 216 (21.5) | 201 (22.4) | 44 (19.1) | 9 (10.3) | 6 (35.3) | 3 (15.8) | 9 (19.6) |
| Dialysis, n (%) | 111 (11.1) | 103 (11.5) | 21 (9.1) | 7 (8.0) | 1 (5.9) | 2 (10.5) | 2 (4.3) |
| Re-Operation, n (%) | 272 (27.1) | 255 (28.4) | 47 (20.4) | 11 (12.6) | 6 (35.3) | 4 (21.1) | 8 (17.4) |

**Supplementary Table 4:** Adverse events in unmatched patients. CAD = coronary artery disease, PCI = percutaneous coronary intervention, CABG = coronary artery bypass graft.

| **Cause of death in unmatched patients without and with relevant CAD** | | | | |
| --- | --- | --- | --- | --- |
| **Characteristics** | **Overall (n = 1003)** | **Patients without  CAD**  **(n =899)** | **Patients with CAD**  **(n =104)** | **p-value** |
| **Cause of Death** | | | | |
| Bleeding, n (%) | 35 (7.1) | 33 (7.4) | 2 (3.9) | 0.563 |
| Chronic lung allograft dysfunction, n (%) | 97 (19.6) | 86 (19.4) | 11 (21.6) | 0.711 |
| Cardiovascular, n (%) | 41 (8.3) | 34 (7.7) | 7 (13.7) | 0.174 |
| Infection/Sepsis, n (%) | 131 (26.5) | 114 (25.7) | 17 (33.3) | 0.245 |
| Malignancy, n (%) | 46 (9.3) | 41 (9.3) | 5 9.8) | 0.803 |
| Multi-organ failure, n (%) | 41 (8.3) | 37 (8.4) | 4 (7.8) | >0.999 |
| Mors in tabula, n (%) | 5 (1.0) | 4 (0.9) | 1 (2.0) | 0.421 |
| Other, n (%) | 31 (6.3) | 29 (6.5) | 2 (3.9) | 0.760 |
| Unknown, n (%) | 67 (13.6) | 65 (14.7) | 2 (3.9) | 0.031 |

**Supplementary Table 5:** Cause of death in unmatched patients without and with relevant CAD. CAD = coronary artery disease.

| **Cause of death in unmatched patients** | | | | | | | |  |
| --- | --- | --- | --- | --- | --- | --- | --- | --- |
| **Characteristics** | **Overall (n = 1003)** | **Patients without  CAD**  **(n =899)** | **Patients with coronary sclerosis**  **(n = 230)** | **Patients with one or two vessel disease**  **(n = 87)** | **Patients with three vessel disease**  **(n = 17)** | **Patients with prior myocardial infarction**  **(n = 19)** | **Patients with prior revascularization**  **(n = 46)** |  |
| **Cause of Death** | | | | | | | | |
| Bleeding, n (%) | 35 (7.1) | 33 (7.4) | 8 (3.5) | 2 (2.3) | 0 (0.0) | 0 (0.0) | 1 (2.2) |  |
| BOS, n (%) | 97 (19.6) | 86 (19.4) | 21 (9.1) | 10 (11.5) | 1 (5.9) | 1 (5.3) | 4 (8.7) |  |
| Cardiovascular, n (%) | 41 (8.3) | 34 (7.7) | 14 (6.1) | 6 (6.9) | 1 (5.9) | 1 (5.3) | 4 (8.7) |  |
| Infection/Sepsis, n (%) | 131 (26.5) | 114 (25.7) | 33 (14.3) | 15 (17.2) | 2 (11.8) | 5 (26.3) | 6 (13.0) |  |
| Malignancy, n (%) | 46 (9.3) | 41 (9.3) | 11 (4.8) | 4 (4.6) | 1 (5.9) | 2 (10.5) | 4 (8.7) |  |
| Multi-organ failure, n (%) | 41 (8.3) | 37 (8.4) | 11 (4.8) | 4 (4.6) | 0 (0.0) | 1 (5.3) | 2 (4.3) |  |
| Mors in tabula, n (%) | 5 (1.0) | 4 (0.9) | 1 (0.4) | 1 (1.1) | 0 (0.0) | 0 (0.0) | 0 (0.0) |  |
| Other, n (%) | 31 (6.3) | 29 (6.5) | 6 (2.6) | 1 (1.1) | 1 (5.9) | 0 (0.0) | 2 (4.3) |  |
| Unknown, n (%) | 67 (13.6) | 65 (14.7) | 14 (6.1) | 2 (2.3) | 0 (0.0) | 0 (0.0) | 1 (2.2) |  |

**Supplementary Table 6:** Cause of death in unmatched patients. CAD = coronary artery disease, BOS = bronchiolitis obliterans syndrome.
